# DNA methylation transcriptionally regulates the putative tumor cell growth suppressor *ZNF677* in non-small cell lung cancers

**DOI:** 10.18632/oncotarget.2697

**Published:** 2014-12-05

**Authors:** Gerwin Heller, Corinna Altenberger, Bianca Schmid, Maximilian Marhold, Erwin Tomasich, Barbara Ziegler, Leonhard Müllauer, Christoph Minichsdorfer, György Lang, Adelheid End-Pfützenreuter, Balazs Döme, Britt-Madeleine Arns, Kwun M. Fong, Casey M. Wright, Ian A. Yang, Walter Klepetko, Christoph C. Zielinski, Sabine Zöchbauer-Müller

**Affiliations:** ^1^ Department of Medicine I, Clinical Division of Oncology, Medical University of Vienna, Vienna, Austria; ^2^ Comprehensive Cancer Center, Medical University of Vienna, Vienna, Austria; ^3^ Department of Pathology, Medical University of Vienna, Vienna, Austria; ^4^ Division of Thoracic Surgery, Medical University of Vienna, Vienna, Austria; ^5^ Department of Thoracic Surgery, National Institute of Oncology-Semmelweis University, Budapest, Hungary; ^6^ Department of Thoracic Oncology and Tumor Biology, National Koranyi Institute of Pulmonology, Budapest, Hungary; ^7^ Landesklinikum Thermenregion Hochegg, Grimmenstein, Austria; ^8^ University of Queensland, The Prince Charles Hospital, Queensland, Chermside, Australia

**Keywords:** DNA methylation, microarray analysis, *ZNF677*, MS-HRM analysis, non-small cell lung cancer

## Abstract

In our study, we investigated the role of *ZNF677* in non-small cell lung cancers (NSCLC). By comparing *ZNF677* expression in primary tumor (TU) and in the majority of cases also of corresponding non-malignant lung tissue (NL) samples from > 1,000 NSCLC patients, we found tumor-specific downregulation of *ZNF677* expression (adjusted *p*-values < 0.001). We identified methylation as main mechanism for *ZNF677* downregulation in NSCLC cells and we observed tumor-specific *ZNF677* methylation in NSCLC patients (*p* < 0.0001). In the majority of TUs, *ZNF677* methylation was associated with loss of ZNF677 expression. Moreover, ZNF677 overexpression in NSCLC cells was associated with reduced cell proliferation and cell migration. ZNF677 was identified to regulate expression of many genes mainly involved in growth hormone regulation and interferon signalling. Finally, patients with *ZNF677* methylated TUs had a shorter overall survival compared to patients with *ZNF677* not methylated TUs (*p* = 0.013). Overall, our results demonstrate that *ZNF677* is trancriptionally regulated by methylation in NSCLCs, suggest that ZNF677 has tumor cell growth suppressing properties in NSCLCs and that *ZNF677* methylation might serve as prognostic parameter in these patients.

## INTRODUCTION

Gene expression in malignant tumors may be affected by genetic and epigenetic changes. In non-small cell lung cancers (NSCLC) many genes are already known whose function is influenced by genetic abnormalities and some of them are also of clinical relevance [1–3]. DNA methylation (referred to as methylation) which is an epigenetic change was identified as alternative mechanism to transcriptionally regulate expression of certain genes. Methylation describes the covalent addition of a methyl group to the 5′ carbon of cytosine bases within cytosine-guanine (CpG) dinucleotides located at high density in CpG islands (CGI) leading to transcriptional gene silencing [4]. It may be reversible by DNA methyltransferase inhibitors like 5-aza-2′-deoxycytidine (Aza-dC) and a synergistic effect in upregulation of gene expression together with histone deacetylase inhibitors like trichostatin A (TSA) was described [5, 6].

So far, many genes were found which are transcriptionally inactivated by methylation in NSCLCs [7–12]. We recently identified ~500 tumor-specifically methylated genes when we used a genome-wide approach to search for methylated genes in NSCLC patients [7].

In addition, to obtain information about expression of these genes, we used publicly available gene expression microarray data of primary tumor (TU) and corresponding non-malignant lung tissue (NL) samples of a large number of NSCLC patients [13, 14]. Many genes which have been identified in our study to be tumor-specifically methylated exhibited downregulated expression in TU compared to NL samples of these patients. One of them is the zink finger protein 677 (*ZNF677*). This gene is located at the chromosomal region 19q13, a region where frequent loss of heterozygosity in NSCLCs occurs [15, 16]. Because our initial results suggested that *ZNF677* is involved in the pathogenesis of NSCLCs and only little information about this gene is currently available, we decided to further investigate this gene. Thus, we determined *ZNF677* mRNA expression, methylation and re-expression in NSCLC cell lines, we investigated *ZNF677* methylation in clinical samples of a large number of NSCLC patients and we compared *ZNF677* methylation with ZNF677 protein expression in tissue samples of some of these patients. In addition, we analysed tumor cell growth suppressing properties of ZNF677 and determined molecular pathways which are affected by ZNF677. Finally, we compared *ZNF677* methylation results of TU samples with clinico-pathological characteristics of the NSCLC patients.

Overall, our results indicate that *ZNF677* is frequently transcriptionally silenced by methylation in NSCLCs and they suggest that *ZNF677* has tumor cell growth suppressing properties. Moreover, *ZNF677* methylation might be of prognostic relevance for NSCLC patients.

## RESULTS

### *ZNF677* expression in clinical samples of NSCLC patients using ArrayExpress and TCGA datasets

**Figure 1:**
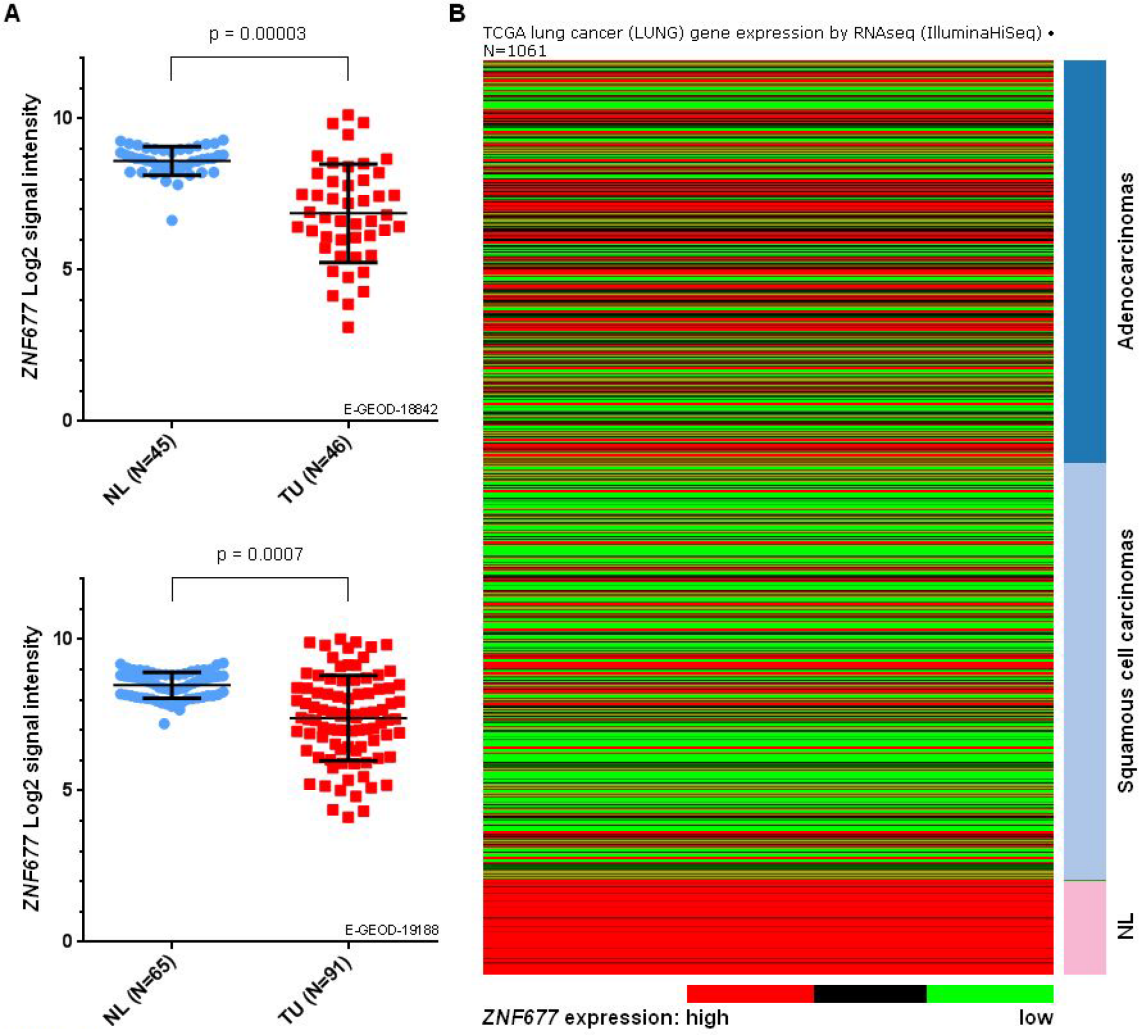
Comparison of *ZNF677* expression values based on gene expression microarray data and RNA-seq data of NL and primary TU samples from NSCLC patients **(A)** Log2 transformed *ZNF677* expression values of 2 independent Affymetrix HG-U133_plus_2.0 datasets are shown. Each dot represents a unique tissue sample. NL, non-malignant lung tissue; TU, primary tumor. **(B)** The heatmap summarises RNA-seq data of *ZNF677* in 1061 clinical samples of NSCLC patients obtained from LUAD and LUSC datasets. Dark blue, adenocarcinomas; light blue, squamous cell carcinomas.

We analysed 2 publicly available microarray datasets from ArrayExpress database to investigate *ZNF677* expression in TU and NL samples of NSCLC patients. While *ZNF677* expression was observed in all NL samples, downregulated *ZNF677* expression was found in TU samples of both datasets analysed (Figure 1A). A statistically significant downregulation of *ZNF677* expression in TU compared to NL samples was observed in dataset E-GEOD-18842 (Bonferroni adjusted *p*-value = 0.00003) and similar results were seen in dataset E-GEOD-19188 (Bonferroni adjusted *p*-value = 0.0007).

To confirm tumor-specific downregulation of *ZNF677* expression observed by gene expression microarray analyses, we additionally compared *ZNF677* RNA-seq expression values in TU and NL samples of LUAD and LUSC datasets obtained from the Cancer Browser database [17]. Consistent to the data obtained by gene expression microarray analyses, a statistically significant tumor-specific downregulation of *ZNF677* expression in NSCLC patients (*p* = 2.7 * 10^−21^) was found (Figure 1B).

We also investigated if downregulation of *ZNF677* expression differs between NSCLC subtypes “adenocarcinoma” (ADC), “squamous cell carcinoma” (SCC) and “large cell carcinoma”. While in dataset E-GEOD-18842 *ZNF677* expression was lower in SCC samples compared to ADC samples (*p* = 0.004), no statistically significant differences of *ZNF677* expression between NSCLC subtypes were observed in dataset E-GEOD-19188 and in the TCGA NSCLC datasets.

### *ZNF677* expression in other tumor entities using TCGA datasets

Because we were interested if *ZNF677* expression is also deregulated in tumors of other entities, we analysed *ZNF677* expression in primary TU and non-malignant tissue samples from patients with other malignancies than NSCLC using TCGA RNA-seq datasets from the Cancer Browser database [17]. We found tumor-specific *ZNF677* downregulation in “breast invasive carcinoma” (*p* = 8.2 * 10^−46^), “colon and rectum adenocarcinoma” (*p* = 1.5 * 10^−7^), “kidney renal clear cell carcinoma” (*p* = 1 * 10^−20^), “head and neck squamous cell carcinoma” (*p* = 1 * 10^−11^) and “uterine corpus endometrioid carcinoma” (*p* = 0.001) patients (supplementary Figure S1).

Overall, these data indicate that *ZNF677* expression is tumor-specifically downregulated in different solid tumor types including NSCLCs.

### *ZNF677* expression in NHBECs and in NSCLC cell lines

**Figure 2:**
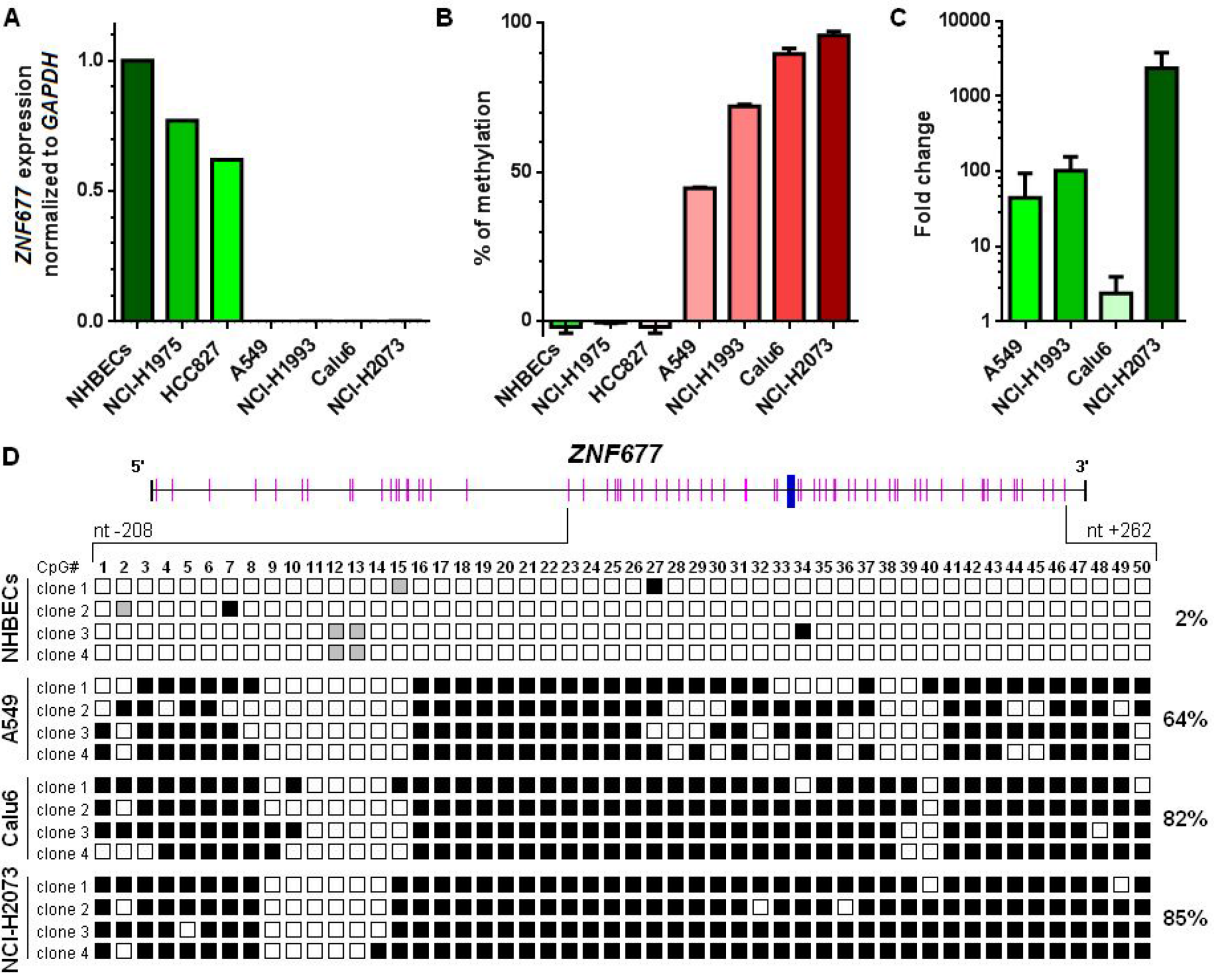
*ZNF677* expression and *ZNF677* methylation in NHBECs and in NSCLC cells **(A)** *ZNF677* expression was determined by RT-PCR using Taqman assays and normalised to *GAPDH*. **(B)** *ZNF677* methylation determined by MS-HRM analyses in NHBECs and in 6 NSCLC cell lines. All NSCLC cell lines found to be negative for *ZNF677* expression were found to be *ZNF677* methylated. **(C)** A549, NCI-H1993, Calu6 and NCI-H2073 cells were treated with a combination of Aza-dC and TSA. *ZNF677* expression was found to be upregulated in all drug treated cells determined by RT-PCR. The fold change in expression of drug treated cells compared to untreated cells is shown. **(D)** Results from BGS of a part of the *ZNF677* 5´ region in NHBECs and in A549, Calu6 and NCI-H2073 cells are demonstrated. Overall, 50 CpG sites (pink bars) were analysed for methylation. The transcription start site is shown as blue bar. Four clones per sample were sequenced. Black squares indicate methylated cytosines at CpG sites, white squares indicate unmethylated cytosines at CpG sites and grey squares indicate CpG sites which were not evaluable.

To further investigate *ZNF677* expression, we analysed NHBECs and NSCLC cell lines A549, Calu-6, HCC827, NCI-H1975, NCI-H1993 and NCI-H2073 for *ZNF677* expression by RT-PCR. *ZNF677* expression was observed in NHBECs and in HCC827 and NCI-H1975 cells, but was not detected in A549, Calu-6, NCI-H1993 and NCI-H2073 cells (Figure 2A). *GAPDH* expression was seen in all samples at comparable Ct values.

### *ZNF677* methylation in NHBECs and in NSCLC cell lines

To study the mechanism responsible for *ZNF677* silencing in many NSCLC cell lines, we developed a MS-HRM assay to determine methylation of the 5′ region of *ZNF677*. As expected, NHBECs were found to be not *ZNF677* methylated (Figure 2B). While all *ZNF677* not expressing cell lines (A549, Calu6, NCI-H1993 and NCI-H2073) were identified to be *ZNF677* methylated, the 2 *ZNF677* expressing cell lines HCC827 and NCI-H1975 were found to be not *ZNF677* methylated. In addition, to demonstrate that methylation of *ZNF677* is indeed associated with its transcriptional regulation, we treated cells of *ZNF677* methylated cell lines A549, Calu6, NCI-H1993 and NCI-H2073 with Aza-dC and TSA and compared *ZNF677* expression in treated and untreated cells by RT-PCR. *ZNF677* expression was found to be upregulated in cells of all drug treated NSCLC cell lines analysed with mean fold changes in expression ranging from 2.5-fold to 2349-fold (Figure 2C).

To confirm data obtained by MS-HRM analyses, we additionally performed BGS of a part of the 5′ region of *ZNF677* in NHBECs and in A549, Calu6 and NCI-H2073 cells. Genomic regions selected for BGS contained regions analysed by MS-HRM. In total, 50 CpG sites were analysed for *ZNF677* methylation (Figure 2D). While in A549, Calu6 and NCI-H1975 cells 64%, 82% and 85% of CpG sites analysed were found to be *ZNF677* methylated, respectively, in NHBECs only 2% of CpG sites were found to be *ZNF677* methylated. Differences in methylation of *ZNF677* in A549 cells and NHBECs, in Calu6 cells and NHBECs and in NCI-H2073 cells and NHBECs were statistically significant (Bonferroni adjusted *p* < 0.001).

### *ZNF677* methylation in cell lines of other tumor entities

In addition, we analysed *ZNF677* methylation in cell lines of other tumor entities including breast cancer (*N* = 5), colon cancer (*N* = 2), ovarian cancer (*N* = 2), pancreatic cancer (*N* = 2) and head and neck cancer (*N* = 2) by MS-HRM (supplementary Figure S2). All these tumor cell lines except 1 breast cancer (MDA-MB-468) and 1 ovarian cancer (SCOV3) cell line were found to be *ZNF677* methylated. By comparing methylation values of these cell lines with *ZNF677* mRNA expression values from the TCGA Cancer Cell Line Encyclopedia, a strong negative correlation between *ZNF677* methylation and *ZNF677* expression in these cell lines was observed (*R* = −0.889, *p* < 0.0001; supplementary Figure S2).

Overall, these results suggest that methylation of the 5´ region of *ZNF677* is responsible for *ZNF677* silencing in many tumor cell lines of different entities including NSCLC, breast cancer, colon cancer, ovarian cancer, pancreatic cancer and head and neck cancer.

### *ZNF677* methylation in NSCLC patients

**Figure 3:**
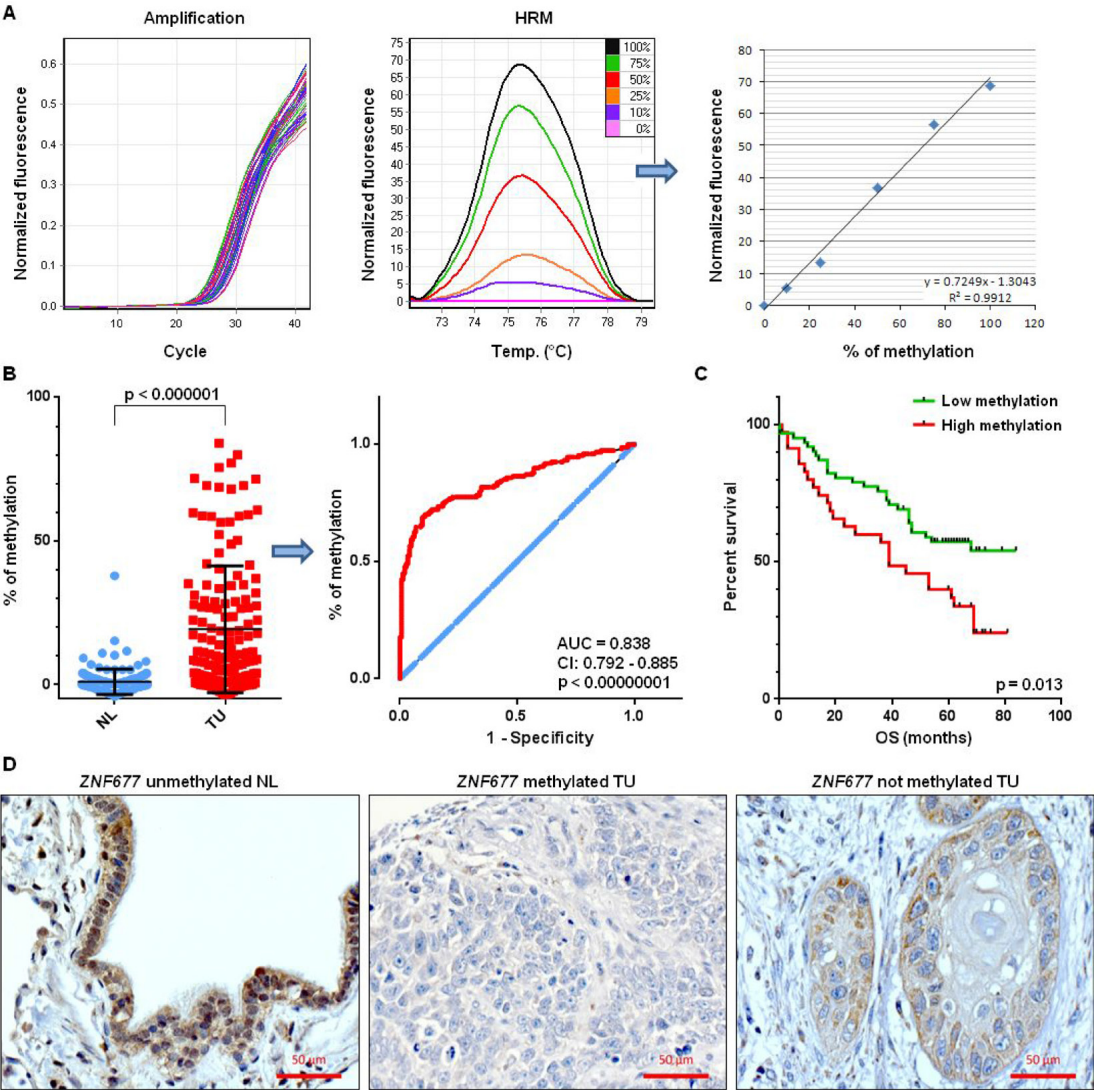
*ZNF677* methylation and ZNF677 expression in clinical samples of NSCLC patients **(A)** A part of the *ZNF677* 5´ region was amplified (left), PCR products were subjected to high resolution melting and fluorescence values were normalised against 0% methylated DNA standards (middle). The regression line of maximum peak values was used for calculation of methylation percentages (right). **(B)** Comparison of % of *ZNF677* methylation in TU and NL samples of 147 NSCLC patients reveals statistically significant differences of *ZNF677* methylation. Each dot represents the % of methylation of an individual sample (left). ROC curve analysis of *ZNF677* methylation in TU and NL samples of 147 NSCLC patients is illustrated (middle). Blue line, reference line; red line, methylation of particular gene; AUC, area under the curve; CI, confidence interval. **(C)** The impact of *ZNF677* methylation on the OS of 97 NSCLC patients is shown. Kaplan-Meier plot demonstrates a statistically significant shorter OS of NSCLC patients with high *ZNF677* methylation compared to NSCLC patients with low *ZNF677* methylation. **(D)** Immunohistochemical staining of a NL sample (left), of a *ZNF677* methylated TU sample (middle) and of a *ZNF677* not methylated TU sample (right) using an antibody to ZNF677 is shown. While nuclear and cytoplasmic ZNF677 expression was observed in bronchial and bronchiolar cells of the NL sample, lack of ZNF677 expression was observed in *ZNF677* methylated tumor cells.

We also investigated *ZNF677* methylation in TU and NL samples of 147 stage I-III NSCLC patients by MS-HRM (Figure 3A). Differences in methylation between TU and NL samples were statistically significant (*p* = 1.3 * 10^−20^) demonstrating that *ZNF677* is tumor-specifically methylated (Figure 3B). The mean % of *ZNF677* methylation was 19 (range −4% – 84%) in TU and 1 (range −4% – 38%) in NL samples, respectively (Figure 3B). In addition, ROC curve analyses revealed that methylation of *ZNF677* statistically significant distinguishes TU from NL samples (*p* = 1.1 * 10^−23^; area under the curve: 0.838; 95% CI: 0.792 – 0.885; Figure 3B).

For comparison of *ZNF677* methylation data with clinico-pathological characteristics of the NSCLC patients, patients were grouped into “high *ZNF677* TU methylation” and “low *ZNF677* TU methylation” using the mean % of *ZNF677* methylation in TU samples as cut-off level [18]. Data about DFS and OS were available for 97 patients, respectively. Patients with “high *ZNF677* TU methylation” had a statistically significant shorter OS than patients with “low *ZNF677* TU methylation” in univariate analyses (median survival: 39 months vs. not reached, *p* = 0.013; Figure 3C). In addition, multivariate analyses identified *ZNF677* TU methylation as independent prognostic factor for shorter OS of NSCLC patients (hazard ratio [HR] = 1.8, 95% confidence interval [CI] = 1 to 3.1, *p* = 0.046). No other statistically significant associations between *ZNF677* methylation and clinico-pathological characteristics of the patients were observed. We also compared *ZNF677* mRNA expression in TU samples and OS of patients in the LUAD and LUSC datasets and, additionally, in a combined version of these 2 datasets (LUAD + LUSC). While we found a shorter OS for patients with *ZNF677* low expressing tumors compared to *ZNF677* high expressing tumors in LUAD (*p* = 0.015) and in the LUAD + LUSC (*p* = 0.029) datasets, we did not find a correlation in the LUSC dataset.

Overall, these results demonstrate that *ZNF677* is tumor-specifically methylated in NSCLC patients. Moreover, they suggest that *ZNF677* methylation and *ZNF677* mRNA expression might be of prognostic impact for certain NSCLC patients, however, additional studies are necessary to determine the potential clinical relevance.

### IHC

We also performed IHC of ZNF677 in FFPE TU samples of a subgroup of 35 NSCLC patients which were also analysed by MS-HRM. A NL sample was used as positive control and nuclear and cytoplasmic ZNF677 expression was observed in bronchial and bronchiolar epithelial cells of this sample (Figure 3D). 43% of the TU samples were scored as negative for ZNF677 protein expression by IHC. Representative stainings are shown in Figure 3D.

In addition, we compared *ZNF677* methylation and ZNF677 protein expression in TU samples. In the majority of TU samples the detection of *ZNF677* methylation was associated with downregulated ZNF677 protein expression. In addition, TU samples which were found to be not *ZNF677* methylated mostly expressed ZNF677. However, these results did not reach statistical significance. Thus, we compared methylation and expression data from LUAD and LUSC datasets and we found a strong negative correlation between *ZNF677* methylation and *ZNF677* expression in both datasets (*R* = −0.673 and *R* = −0.654, respectively; *p* < 0.000001, respectively; supplementary Figure S2C).

### *ZNF677* mutations and copy number changes in NSCLC patients

Moreover, we were interested if besides methylation also mutations or copy number changes might be responsible for downregulated *ZNF677* expression in NSCLC patients. Thus, we searched for *ZNF677* mutations in LUAD and LUSC datasets. *ZNF677* mutations including 1 splice variant and 6 missense mutations (C232S, H477N, G254W, C492F, H281Y and P514T) were found only in 2% of both ADC and SCC patients and their localisation is shown in supplementary Figures S3A and S3B. All *ZNF677* mutations were mutually exclusive. Mutations H477N and C492F were predicted to have a high functional impact (supplementary Figure S3C).

In addition, we searched for *ZNF677* copy number changes in LUAD and LUSC TCGA aCGH datasets of 983 NSCLC patients. A homozygous *ZNF677* deletion was detected only in 1 ADC patient (supplementary Figures S3D and S3E), however, heterozygous *ZNF677* deletions were found in 34% of ADC patients and in 25% of SCC patients. Overall, the pattern of *ZNF677* copy number aberrations was comparable to that of other genes known to be frequently inactivated by methylation in NSCLC patients (e.g. *p16*, *RASSF1A*, *DAPK1*, *DAL-1*, *CDH1*; supplementary Figures S3D and S3E) [8, 9, 19]. Copy number alterations did not correlate with changes in *ZNF677* expression.

These results demonstrate that heterozygous *ZNF677* deletions but not *ZNF677* mutations occur frequently in NSCLCs indicating that *ZNF677* mutations do not play a role in inactivation of *ZNF677* in NSCLCs.

### Effect of ZNF677 expression on cell viability, cell proliferation, apoptosis and cell migration

**Figure 4:**
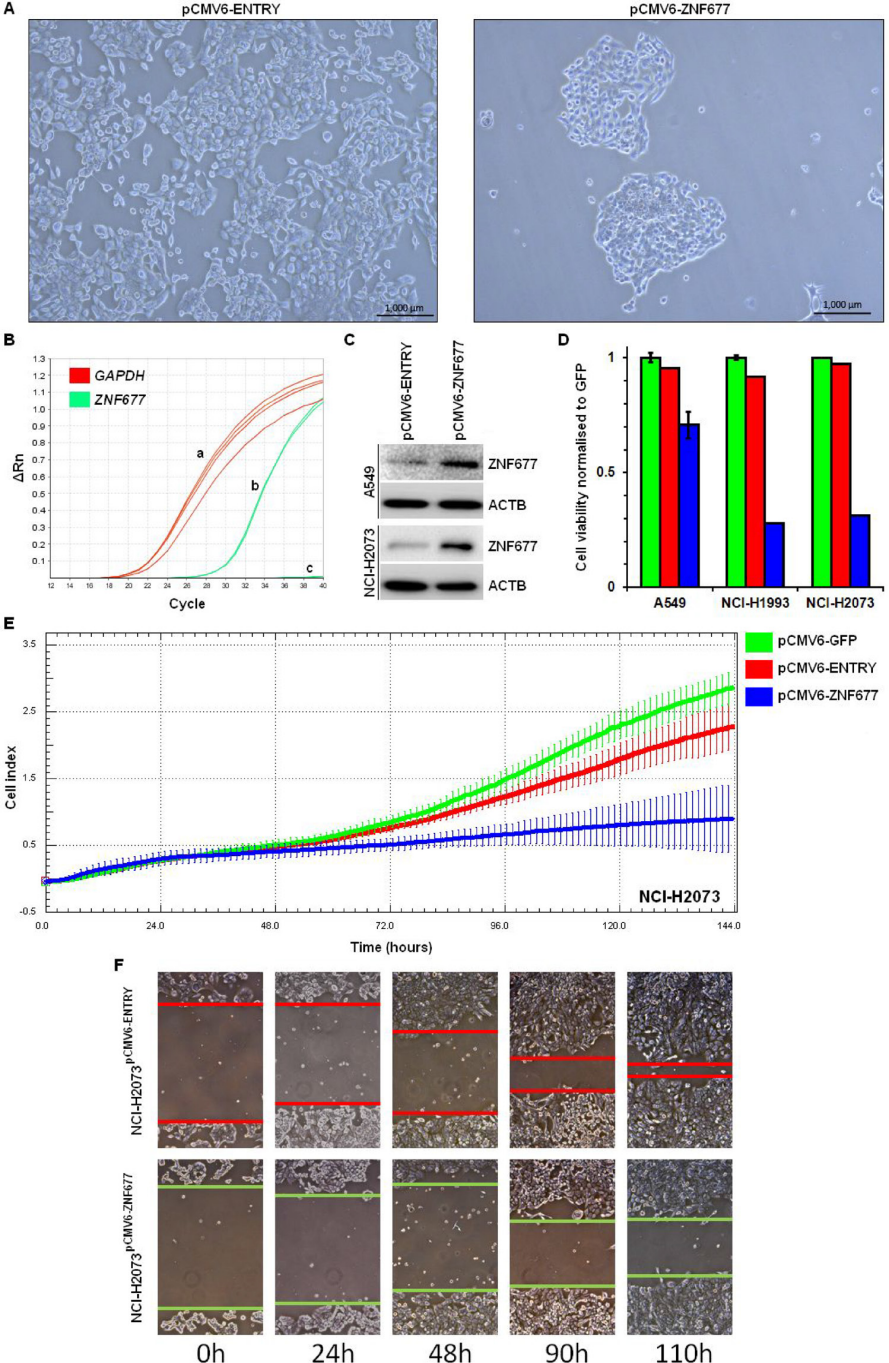
Cell viability, cell proliferation and cell migration of pCMV6-ZNF677 transfected NSCLC cells **(A)** NCI-H1993 ^pCMV6-ENTRY^ cells were densely grown after 4 weeks of G418 treatment (left), however, reduced growth of NCI-H1993 ^pCMV6-ZNF677^ cells was observed (right). **(B)** While GAPDH expression was detected in all samples analysed (a), *ZNF677* was detected in pCMV6-ZNF677 transfected cells (b) but not in control cells (c) by RT-PCR. **(C)** ZNF677 overexpression confirmed by Western blotting in pCMV6-ENTRY and pCMV6-ZNF677 transfected A549 and NCI-H2073 cells. **(D)** Reduced cell viability of pCMV6-ZNF677 transfected A549, NCI-H1993 and NCI-H2073 cells compared to control cells was observed. Experiments were performed in triplicates. Error bars indicate standard deviations. **(E)** Reduced cell proliferation of pCMV6-ZNF677 transfected A549, NCI-H1993 and NCI-H2073 cells compared to controls using xCELLigence RTCA system was observed. Each cell line was plated in triplicates. Error bars indicate standard deviations. **(F)** Scratch assay of NCI-H2073^pCMV6-ENTRY^ and NCI-H2073^pCMV6-ZNF677^ cells demonstrates a faster closure of the scratch in NCI-H2073^pCMV6-ENTRY^ cells compared to NCI-H2073^pCMV6-ZNF677^ cells.

To investigate the biologic effect of ZNF677 expression in NSCLC cells, we stably transfected A549, NCI-H2073 and NCI-H1993 cells with a pCMV6-ZNF677 expression vector, with a pCMV6-ENTRY (empty vector) and with a pCMV6-GFP control vector. Using light microscopy, we observed strongly reduced cell growth in pCMV6-ZNF677 transfected cells compared to empty vector control cells (Figure 4A). ZNF677 overexpression was confirmed by RT-PCR and Western blotting (Figure 4B and 4C). Reduced cell growth (1.4 to 3.6-fold, mean 2.7-fold) of pCMV6-ZNF677 transfected A549, NCI-H2073 and NCI-H1993 cells was confirmed using a cell viability assay (Figure 4D). In addition, cell proliferation of transfected cells was measured over time and reduced cell proliferation of pCMV6-ZNF677 transfected cells compared to control cells was observed confirming our cell viability assay results (Figure 4E).

No effect of ZNF677 expression on Caspase 3 activity was observed (data not shown). However, we observed a reduced migration capacity of pCMV6-ZNF677 transfected cells compared to cells transfected with the empty control vector by using a scratch assay (Figure 4F).

Overall, these findings suggest that ZNF677 affects cell proliferation and migration of NSCLC cells A549, NCI-H1993 and NCI-H2073.

### Transcriptional gene regulation by ZNF677

**Figure 5:**
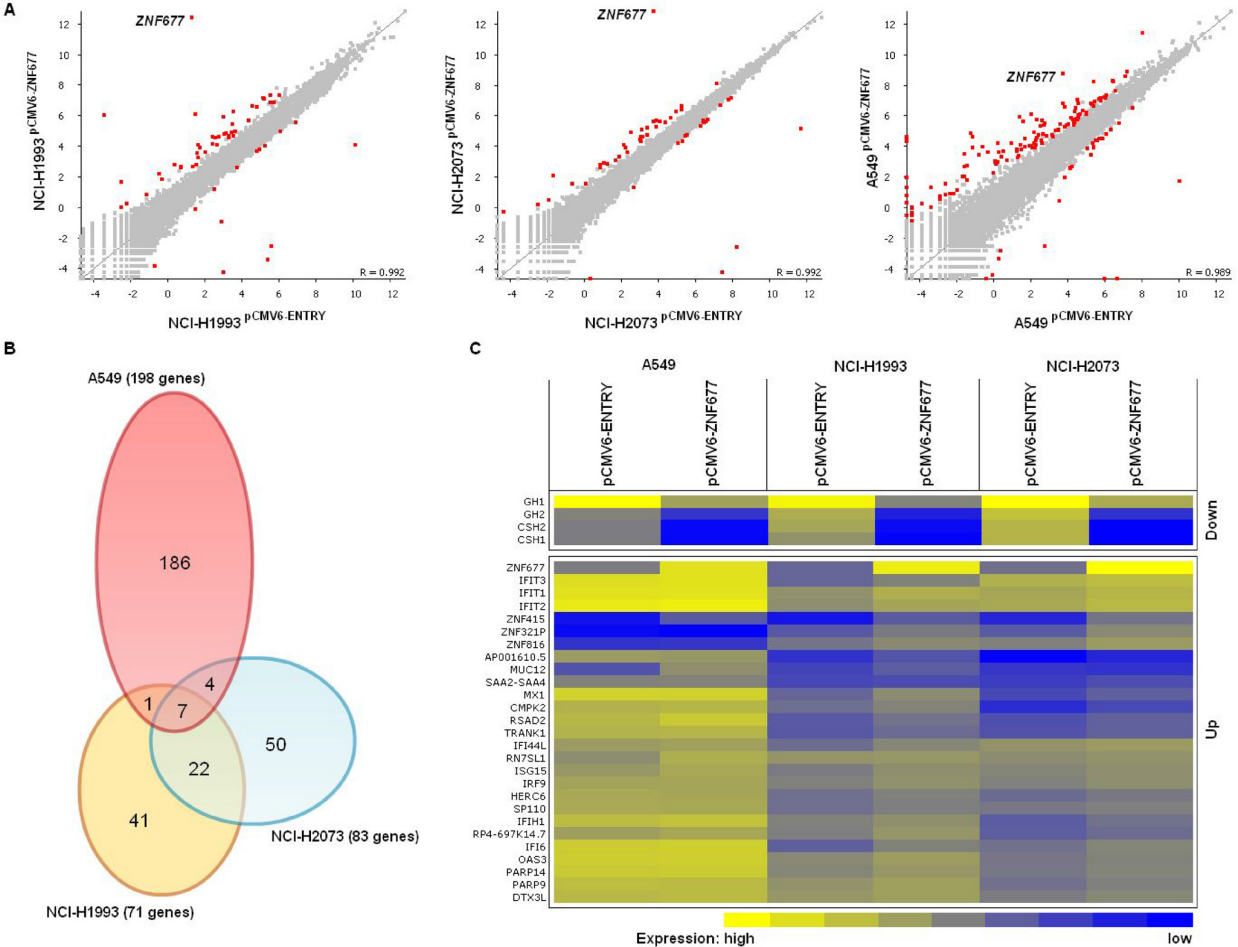
Effect of ZNF677 overexpression on the transcriptome of NSCLC cells **(A)** Scatter plots showing ZNF677 regulated genes in pCMV6-ZNF677 transfected A549, NCI-H1993 and NCI-H2073 cells compared to control cells analysed by RNA-seq. Red dots indicate statistically significant deregulated genes. **(B)** The venn diagram summarizes the overlap of ZNF677 regulated genes in A549, NCI-H1993 and NCI-H2073 cells. **(C)** The heatmap shows expression values of 34 genes found to be either up- or downregulated in at least 2 pCMV6-ZNF677 transfected NSCLC cell lines compared to control cells. Expression values range from yellow (high) to blue (low).

Finally, we determined the effect of ZNF677 expression on the transcriptome of A549, NCI-H1993 and NCI-H2073 cells by RNA-seq. Overall, ZNF677 overexpression resulted mainly in upregulation of target genes (Figure 5A). We identified 198, 71 and 83 differentially expressed genes in pCMV6-ZNF677 transfected A549, NCI-H1993 and NCI-H2073 cells compared to control cells, respectively (Figure 5B, supplementary Table S1). By comparing differentially expressed genes in the 3 NSCLC cell lines, we identified 34 genes which are regulated by ZNF677 in at least 2 of the cell lines (Figure 5C). Four of these genes were found to be downregulated (*GH1*, *GH2*, *CSH1* and *CSH2*), 27 of them were found to be upregulated and 3 genes were found to be either down- or upregulated. These 34 genes were further categorized based on Gene Ontology (supplementary Figure S4) and protein expression of some of them was analysed by Western blotting (supplementary Figure S5). Several genes whose expression was found to be upregulated in these cells are associated with “immune effector process” (adjusted *p* = 1.7 * 10^−5^), “response to cytokine” (adjusted *p* = 0.002), “type I interferon signaling pathway” (adjusted *p* = 9.4 * 10^−10^) and “response to virus” (adjusted *p* = 1.3 * 10^−8^). Similar results were observed by analysing GO enrichment using genes with induced expression in “*ZNF677* high expressing” compared to “*ZNF677* low expressing” primary TU samples of ArrayExpress datasets E-GEOD-18842 and E-GEOD-19188 (supplementary Table S2).

These results demonstrate that ZNF677 is a transcriptional regulator of genes involved in growth signaling and interferon signaling pathways in NSCLC cells.

## DISCUSSION

In a recent genome-wide search for CGI methylation in a large number of NSCLC patients, we identified 477 tumor-specifically methylated genes and from the majority of them regulation by methylation and/or an involvement in the pathogenesis of NSCLCs was unknown so far [7]. One of these genes is *ZNF677* and except the report from Li et al. who described some genomic aberrations of *ZNF677* in gliomas no manuscripts about the involvement of *ZNF677* in cancer development was published so far [20]. Thus, we decided to further investigate the role of *ZNF677* in NSCLCs.

Large datasets from genome-wide gene expression analyses regarding NSCLCs are publically available [13, 14, 21]. Hou et al. identified 588 unique genes with tumor-specifically downregulated expression by comparing mRNA expression profiles between TU and corresponding NL samples of NSCLC patients using gene expression microarray analyses (E-GEOD-19188) and similar results were reported by Sanchez-Palencia et al. (E-GEOD-18842) [13, 14]. In our study, we used these 2 datasets to search for tumor-specific differences of *ZNF677* expression. After multiple comparison adjustment, we identified *ZNF677* expression to be statistically significantly downregulated in TU compared to NL samples of NSCLC patients in both datasets. Tumor-specific downregulation of *ZNF677* expression in NSCLC patients was confirmed by RNA-seq expression values from the TCGA database [22, 23]. Moreover, we found tumor-specific downregulation of *ZNF677* expression also in breast carcinomas, colon and rectum adenocarcinomas, renal clear cell carcinomas, head and neck squamous cell carcinomas and endometrioid carcinomas suggesting that *ZNF677* may play an important role also in the pathogenesis of several other malignant diseases.

Since we had *ZNF677* identified to be tumor-specifically methylated in our genome-wide methylation analyses before, we used single-gene analyses to confirm methylation of *ZNF677* in several NSCLC cell lines. NSCLC cells which did not express *ZNF677* were found to be *ZNF677* methylated and treatment of these cells with epigenetically active drugs resulted in upregulation of *ZNF677* expression. ZNF677 mutations were found in only 2% of the tumors using TCGA datasets. In addition, we observed an association between *ZNF677* methylation and loss of ZNF677 expression for the majority of TU samples analysed while *ZNF677* not methylated TU samples mostly expressed ZNF677. However, these results did not reach statistical significance which might be explained by the low number of samples available for this analysis. Moreover, we used tissue microarrays from the tumor samples which represent only a small part of the tumor. Because of heterogeneity of tumor cells within a tumor, we cannot exclude that heterogeneity of ZNF677 protein expression might have influenced our results.

However, we were able to demonstrate that downregulated *ZNF677* expression is strongly correlated with increased *ZNF677* methylation in primary TU samples of LUAD and LUSC datasets. Overall, these results indicate that methylation is a major mechanism for transcriptional regulation of *ZNF677*.

We also analysed TU and NL samples of a large number of NSCLC patients and identified statistically significant tumor-specific *ZNF677* methylation. Moreover, ROC curve analyses of MS-HRM results statistically significantly distinguished TU from NL samples. In addition, we compared our *ZNF677* methylation results with certain clinico-pathological characteristics of the NSCLC patients and observed a shorter OS of patients with *ZNF677* methylated tumors compared to patients with *ZNF677* not methylated tumors. These results suggest that *ZNF677* methylation might be of potential prognostic relevance, however, additional studies are necessary to confirm our results. Since we found in an exploratory analysis using LUAD and LUSC datasets that downregulated *ZNF677* expression is associated with a shorter OS in a subgroup of NSCLC patients, also *ZNF677* expression might be of potential prognostic impact. In contrast to some genetic changes which occur only in certain histologic NSCLC subtypes, we did not find a statistically significant difference in *ZNF677* methylation between tumors with squamous and with non-squamous histology [1–3].

Certain characteristics of tumor suppressor genes (TSG) include that they are often located in chromosomal regions with frequent loss of heterozygosity (LOH), that they may become inactivated by genetic or epigenetic mechanisms and that they may inhibit tumor cell growth [24]. *ZNF677* is located at the chromosomal region 19q13 which is frequently affected by LOH in NSCLCs [15, 16]. Moreover, we found ZNF677 to be frequently inactivated in NSCLC cell lines and in primary NSCLCs. Thus, we investigated if ZNF677 has tumor cell growth suppressing properties. Overall, in NSCLC cell line experiments, we observed a strongly reduced tumor cell growth and reduced cell motility of ZNF677 overexpressing cells compared to control cells. Reduced tumor cell growth was observed in all 3 NSCLC cell lines analysed with 3 different assays which suggests that ZNF677 has intrinsic tumor cell growth suppressing function.

Moreover, to uncover molecular pathways affected by *ZNF677* expression in NSCLC cells, we compared the transcriptome of *ZNF677* transfected cells with control cells using RNA-seq technology. The small overlap between A549 cells and cells from the other 2 tumor cell lines (NCI-H1993 and NCI-H2073) might be explained by the different biology of these cells. While A549 is described as a mesenchymal-like cell line, NCI-H1993 and NCI-H2073 are epithelial-like cell lines [25]. All *ZNF677* transfected cell lines demonstrated strongly downregulated expression of genes *GH1*, *GH2*, *CSH1* and *CSH2* which are involved in “JAK-STAT growth hormone signaling pathway” compared to control cells. These highly homologous genes are involved in normal postnatal growth regulation [26]. Interestingly, elevated GH concentrations were found in serum samples of breast cancer patients and an association between autocrine GH1 expression and hyperproliferation of breast cancer cells was reported [27, 28]. However, the role of these growth regulators in NSCLCs is unclear and needs to be determined. In contrast, genes whose expression was seen to be upregulated in ZNF677 transfected cell lines were found to be mainly involved in “type I interferon signaling pathway” and in “response to virus”. While interferon stimulating gene IRF9 is a key factor forcing the anti-proliferative effect of IFN-α, ISG15 is involved in a Wnt/beta-catenin suppressing pathway and may inhibit liver cancer cell growth [29–32]. The function of other interferon stimulated genes like IFIT1, IFIT2 and IFIT3 is yet poorly understood.

Overall, we identified methylation as the major mechanism for frequent transcriptional inactivation of *ZNF677* in NSCLCs. *ZNF677* methylation occurs tumor-specific in NSCLC patients and might be of potential prognostic impact for these patients. Moreover, we observed a strong tumor cell growth suppressing effect of ZNF677 and we identified certain pathways affected by ZNF677 which are involved in regulation of cell proliferation. All these results together indicate that ZNF677 plays an important role in the pathogenesis of NSCLCs.

## MATERIALS AND METHODS

### Tumor cell lines and tissue samples

Lung adenocarcinoma cell lines A549, NCI-H1993 and NCI-H2073 were purchased from the American Type Culture Collection (ATCC) and were stored in liquid nitrogen until use. Lung adenocarcinoma cell lines Calu6, HCC827 and NCI-H1975 were kindly provided by Dr. Walter Berger (Institute of Cancer Research, Medical University of Vienna, Vienna, Austria). Normal human bronchial epithelial cell (NHBECs) pellets were purchased from Promocell. Tumor cells were cultured and treated with a combination of Aza-dC and TSA as reported [8]. Untreated cells were used as controls.

Genomic DNA of breast cancer (BT-20, MCF-7, MDA-MB-231, MDA-MB-453, MDA-MB-468), colon cancer (HCT-15, HT-29), ovarian cancer (SCOV3, A2780), pancreatic cancer (AsPC-1, BxPC-3) and head and neck cancer (Cal-27, FaDu) cell lines was obtained from members of the Medical University of Vienna, Vienna, Austria.

Frozen primary TU and corresponding NL specimens of a convenience sample of 147 stage I, II or III caucasian NSCLC patients who underwent surgical resection of their tumor in a curative intent during the years 2000–2004 were used. Overall, from 97 patients clinico-pathological data including gender, age, histology, tumor stage, lymph node stage, stage of disease, disease recurrence, disease-free survival (DFS) and overall survival (OS) were collected [7]. The median age was 62 years and the median follow-up time was 54 months. None of these 97 patients received adjuvant chemotherapy. This study was approved by the local ethics committee of the MUV. From 35 of these patients also formalin-fixed, paraffin embedded (FFPE) TU and NL samples were available.

### Publicly available datasets

Affymetrix HG-U133_plus_2.0 gene expression microarray data were obtained from ArrayExpress database and the datasets E-GEOD-18842 and E-GEOD-19188 were used [13, 14, 33].

RNA-seq data, Affymetrix SNP array data and Agilent aCGH data were obtained from “The Cancer Genome Atlas” (TCGA) database (http://cancergenome.nih.gov), from cBioPortal for Cancer Genomics (http://www.cbioportal.org) and from Cancer Browser (https://genome-cancer.ucsc.edu/) [34]. The following datasets were used: lung cancer (LUAD and LUSC), colon and rectum adenocarcinoma (COADREAD), kidney clear cell carcinoma (KIRC), head and neck squamous cell carcinoma (HNSC), breast invasive carcinoma (BRCA) and uterine corpus endometrioid carcinoma (UCEC). Caleydo software and Cancer Browser (https://genome-cancer.ucsc.edu) were used for data visualization [17, 35]. A detailed overview about all these datasets is shown in Table 1.

**Table 1:** Summary of NSCLC gene expression microarray datasets obtained from ArrayExpress database and RNA-seq datasets obtained from TCGA / Cancer Browser databases used for *ZNF677* expression analyses

ID	ArrayExpress		TCGA^a^	
	E-GEOD-18842^b^	E-GEOD-19188^c^	LUAD^d^	LUSC^e^
**TU samples (N)**	46	91	470	483
**NL samples (N)**	45	65	58	50
**Matching TU/NL (%)**	96	100	100	100
**Sex**			*N* = 461	*N* = 408
Male (%)	n/a	72	46	75
Female (%)	n/a	28	54	25
**Ethnicity**			*N* = 393	*N* = 317
Caucasian (%)	n/a	89.5	92	92
Asian (%)	n/a	0	2	3
Afro-American (%)	n/a	0	6	5
Other (%)	n/a	4	0	0
Unknown (%)	n/a	6.5	0	0
**Histology (%)**				
ADC	30	49	100	0
SCC	70	30	0	100
LCC	0	21	0	0
**Disease stage (%)**				
I	83	60	54	50
II	9	28	24	30
III	6	9	17	18
IV	2	3	5	2

a Clinical data based on Caleydo software using TCGA datasets version 15.01.2014,

b Sanchez-Palencia et al., Int J Cancer, 2011;

c Hou et al., PLos One, 2010;

d TCGA Research Network, Nature, 2014;

e TCGA Research Network, Nature, 2012. TU, primary tumor; NL, non-malignant lung tissue; ADC, adenocarcinoma; SCC, squamous cell carcinoma; LCC, large cell carcinoma.

### Real-time reverse transcription-PCR (RT-PCR)

Total RNA was isolated from NHBECs and NSCLC cell lines using TRIzol reagent (Invitrogen) and reverse transcribed using OmniScript Reverse Transcriptase Kit (Qiagen). *ZNF677* expression was determined by RT-PCR using Taqman Gene Expression Assays (Applied Biosystems) Hs00737026_m1 (*ZNF677*) and Hs03929097_g1 (*GAPDH*). Differences in gene expression were calculated by standard ΔΔCt method [36].

### Methylation-sensitive high resolution melting (MS-HRM) analyses

Genomic DNA was isolated from NHBECs, tumor cell lines and frozen tissue samples as reported and was stored at −80°C until use for methylation analyses [8]. Afterwards, genomic DNA was modified by treatment with sodium bisulfite using EpiTect Bisulfite kit (Qiagen) [37]. Primers (fwd, 5′-GTTTTTYGGGTTTAAGTTTG-3′ and rev, 5′-AATTTTAACCTACAAAACRACC-3′) were designed based on the genomic *ZNF677* sequence obtained from ENSEMBL database (release 69) using Methyl Primer Express v1.0 software. EpiTect HRM PCR kit in a RotorGene®Q cycler (Qiagen) was used. Methylation standards were constructed by diluting 100% methylated and unmethylated control DNA (Qiagen) at 100%, 75%, 50%, 25%, 10% and 0% ratios [37]. The standards were included in each HRM run performed. All experiments were performed in duplicate.

### Bisulfite genomic sequencing (BGS)

BGS was performed on sodium bisulfite treated genomic DNA as reported previously [7, 37]. Primers (fwd, 5´-TTTAAGGGAATTTAAAAGTGAAGAA-3´ and rev, 5´-TCCACACTAACCTAAAACAAAAAA-3´) were designed using Methyl Primer Express v1.0 software. PCR products were cloned using TOPO^®^ TA Cloning^®^ Kit for Sequencing (Invitrogen). Four clones per cell line (NHBECs, A549, Calu6 and NCI-H2073) were sequenced using M13 primers [7, 37].

### Immunohistochemistry (IHC)

ZNF677 protein expression was determined in FFPE TU and NL samples of 35 NSCLC patients who were also included in our *ZNF677* methylation analysis. A tissue microarray was constructed and used as reported previously [7]. The rabbit polyclonal anti-ZNF677 antibody (1:10, HPA024796, Sigma Aldrich) was used. Results of IHC were scored as no staining (−), weak staining (1+), moderate staining (2+) or strong staining (3+). For comparison of IHC results with methylation results, patients whose TU showed no or weak staining were grouped as “negative by IHC” while patients whose TU showed moderate or strong staining were grouped as “positive by IHC” as reported previously [7].

### Vectors and transfection

pCMV6-ENTRY (empty control, PS100001, Origene) and pCMV6-ZNF677 (RC207997, Origene) were used for transfection experiments. A pCMV6-GFP vector was constructed by subcloning the GFP coding sequence using fwd (5′-GAGGCGATCGCCATGGTGAGCAAGGGCG-3′) and rev (5′-GCGACGCGTCTTGTACAGCTCGTCCATG-3′) primers. Cells were transfected by use of Lipofectamine® LTX reagent (Invitrogen) as recommended by the manufacturer. Stably transfected cells were selected by G418 treatment (Invitrogen) and transfection efficacy was analysed by RT-PCR and Western blotting.

### Western blotting

Protein samples were denatured, separated by SDS/PAGE and transferred onto PVDF membrane (Biorad). Membranes were blocked followed by incubation with primary antibodies anti-ZNF677 (1:100, Abcam), anti-IRF9 (1:50, Santa Cruz Biotechnology), anti-ISG15 (1:1000, Cell Signaling), anti-CSH1 (1:375, Thermo Scientific), anti-ACTB (1:200, Abcam) and anti-GAPDH (0.05mg/ml, Sigma Aldrich). Appropriate secondary HRP antibodies were used and membranes were visualized using ECL Western blotting substrate (Thermo Scientific).

### Cell viability and cell proliferation

CellTiter-Blue® Cell Viability Assay (Promega) was performed as recommended. Cells were seeded in triplicate in 96-well plates and incubated with CellTiter™ Blue reagent prior to fluorescence measurement.

xCELLigence Real-Time Cellular Analysis (RTCA) system (Roche) was used to measure cell proliferation in real time. Cells were seeded in triplicates in 16-well E-plates, cell proliferation was continuously monitored for 1 week and the cell index was recorded every 5 minutes by the RTCA analyzer.

### Caspase assay

Fluorometric caspase assay was performed as described [38, 39]. Cell lysates were incubated with caspase 3 substrate (Enzo) in HEPES buffer. Caspase substrate cleavage was measured at an excitation wavelength of 405 nm and an emission wavelength of 535 nm. Reaction mixture without protein was used for background correction. Cells treated with Doxorubicine were used as positive control.

### Scratch assay

A cell monolayer was scratched with a sterile pipette tip and cells were incubated under standard conditions. The scratch was observed under light microscopy and was photographed at several time points.

### RNA-sequencing (RNA-seq)

Total RNA was prepared from transfected and control cells of NSCLC cell lines using RNeasy kit (Qiagen) and was subjected to RNA-seq according the mRNA sequencing protocol provided by Illumina (TruSeq RNA Sample Preparation Kit) as reported previously [40]. In brief, poly(A)-containing mRNA molecules were purified using poly(T)-oligo-attached magnetic beads, fragmented and applied to first-strand complementary DNA (cDNA) synthesis using reverse transcriptase and random primers. Second-strand cDNA synthesis was performed using DNA polymerase I and RNase H. cDNAs were then end-repaired, A-tailed, ligated to adaptors and amplified to create the final cDNA library. Afterwards, adaptor-ligated cDNA was sequenced on a HiSeq2000 sequencer according to the manufacturer′s instructions.

### Statistical analysis

Raw Affymetrix microarray data were processed and normalised using MAS5 algorithm of Flexarray 1.6 software [41]. Differences in gene expression between tissue types were calculated using ANOVA and resulting *p*-values were adjusted for all probe sets represented on Affymetrix HG-U133_plus_2.0 microarrays (54.675) using Bonferroni method. An adjusted *p*-value < 0.05 was considered as statistically significant.

Wilcoxon signed rank tests were used to calculate *ZNF677* methylation differences between TU and NL samples obtained by MS-HRM analyses. Receiver operating characteristic (ROC) curve analysis was performed using GraphPad Prism 6 software. A *p*-value < 0.05 was considered as statistically significant. Spearman's Rho test was calculated for correlation of methylation and gene expression data from TCGA datasets using R software.

MS-HRM data were compared with clinico-pathological characteristics (gender, age, histology, tumor stage, lymph node stage, stage of disease, disease recurrence, DFS and OS) of NSCLC patients. Chi^2^ tests/Fisher's exact tests were used to calculate differences between groups and t-tests were used to calculate differences between means. Survival analyses of patients were performed using log rank and generalized Wilcoxon testing. A *p*-value < 0.05 was considered as statistically significant. These analyses were performed using the statistics software PASW (version 18).

Galaxy platform and TopHat2 were used to align raw RNA-seq data (fastq files) of *ZNF677* transfected NSCLC cells and of control cells to hg19 [42–45]. Aligned .bam files were imported to SeqMonk v0.27.0 software and processed using RNA-seq quantitation pipeline (http://www.bioinformatics.babraham.ac.uk/projects/seqmonk/). An adjusted *p* < 0.05 was defined as cut-off for differentially expressed genes.

## SUPPLEMENTARY FIGURES AND TABLES


