# Supplementary material for: DNA methylation transcriptionally regulates the putative tumor cell growth suppressor ZNF677 in non-small cell lung cancers
Source: Oncotarget. 2014 Dec 5;6(1):394–408. doi: 10.18632/oncotarget.2697 (PMC4381603; doi:10.18632/oncotarget.2697)
Supplement: Supplementary file 1 [file oncotarget-06-394-s001.pdf]

SUPPLEMENTARY FIGURES AND TABLES

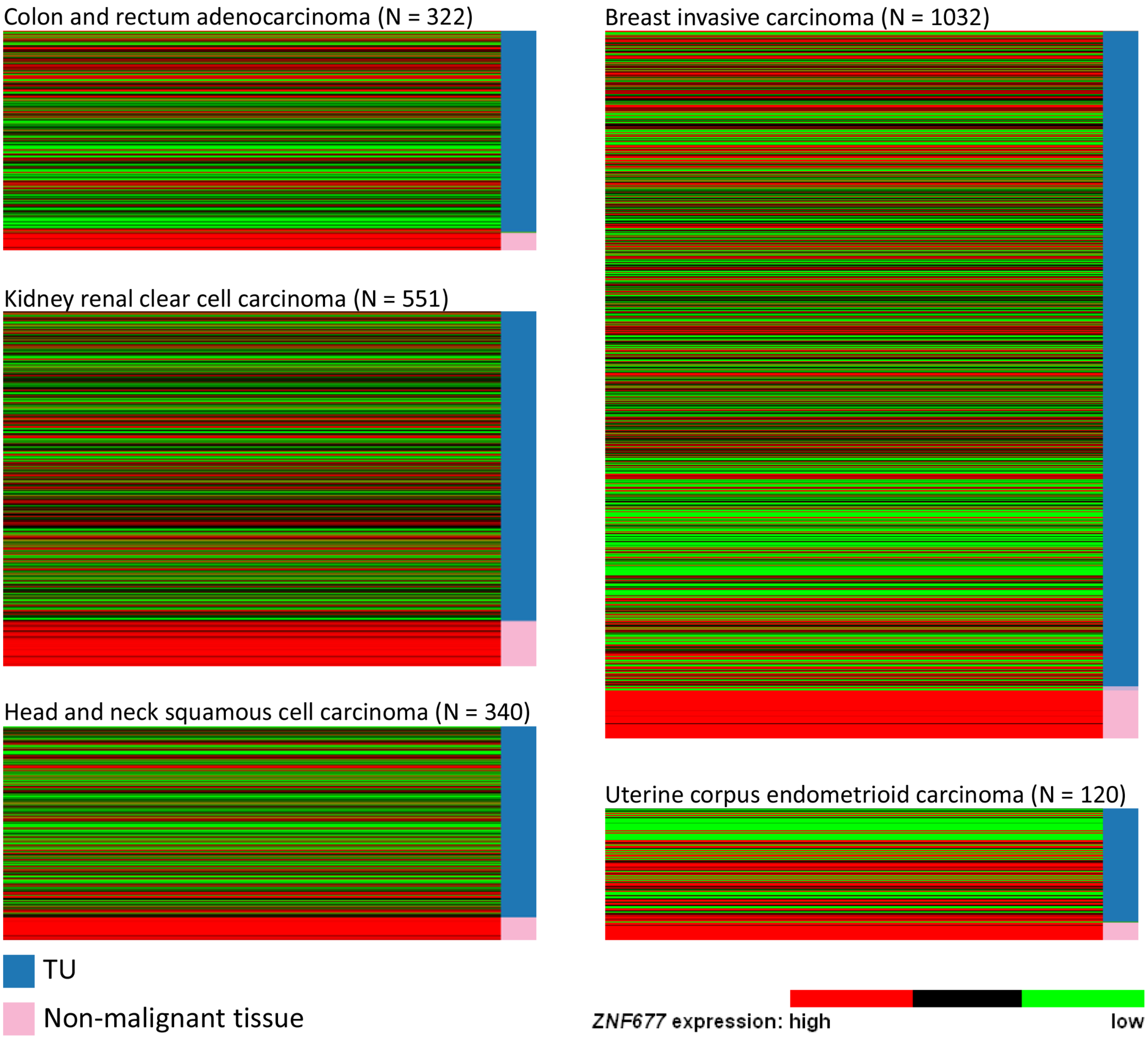

Supplementary Figure S1: Comparison of *ZNF677* gene expression values based on RNA-seq data from TCGA datasets of various TU samples and non-malignant tissue counterparts obtained from cancer browser database (https://genome-cancer.ucsc.edu). Heatmaps summarize RNA-seq data of *ZNF677* in 2365 clinical samples of various cancer patients obtained from TCGA database (COADREAD, KIRC, HNSC, BRCA and UCEC datasets). Each row represents a single patient. Overall, *ZNF677* was consistently expressed in non-malignant tissue samples, however, *ZNF677* expression was downregulated in TU samples. Red, high expression; green, low expression

A

Origin	Cell line	% of methylation
Breast cancer	MDA-MB-468	-2
Breast cancer	BT-20	43
Breast cancer	MDA-MB-231	59
Breast cancer	MCF-7	60
Breast cancer	MDA-MB-453	88
Colon cancer	HCT-15	86
Colon cancer	HT-29	89
Ovarian cancer	SCOV3	-2
Ovarian cancer	A2780	98
Pancreatic cancer	AsPC-1	85
Pancreatic cancer	BxPC-3	88
Head and neck cancer	Cal 27	83
Head and neck cancer	FaDu	99

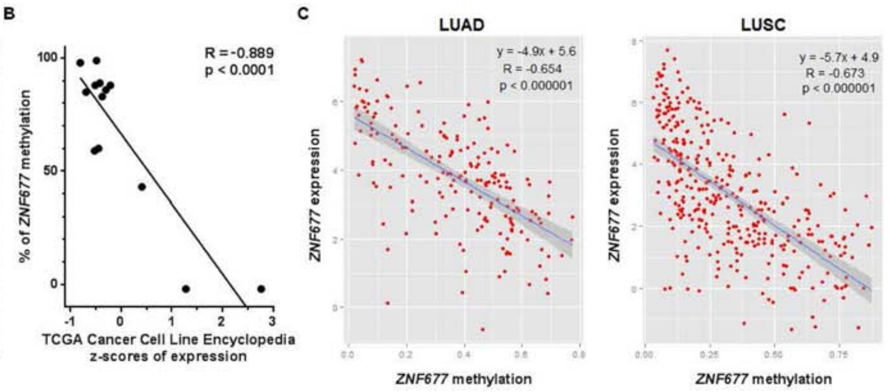

Supplementary Figure S2: (A) Percentages of *ZNF677* methylation in cell lines of various tumor entities analysed using MS-HRM are listed. (B) The Comparison of *ZNF677* methylation in cell lines to *ZNF677* mRNA expression values determined by RNA-seq (Cancer Cell Line Encyclopaedia from TCGA database) is shown. **(C)** The Comparison of *ZNF677* methylation and *ZNF677* expression in primary tumors of 185 lung adenocarcinoma patients (LUAD) and of 351 lung squamous cell carcinoma patients (LUSC) is shown. X-axis, beta values of methylation determined using Illumina Human Methylation 450 BeadChip arrays; y-axis, z-values of expression determined by RNA-seq.

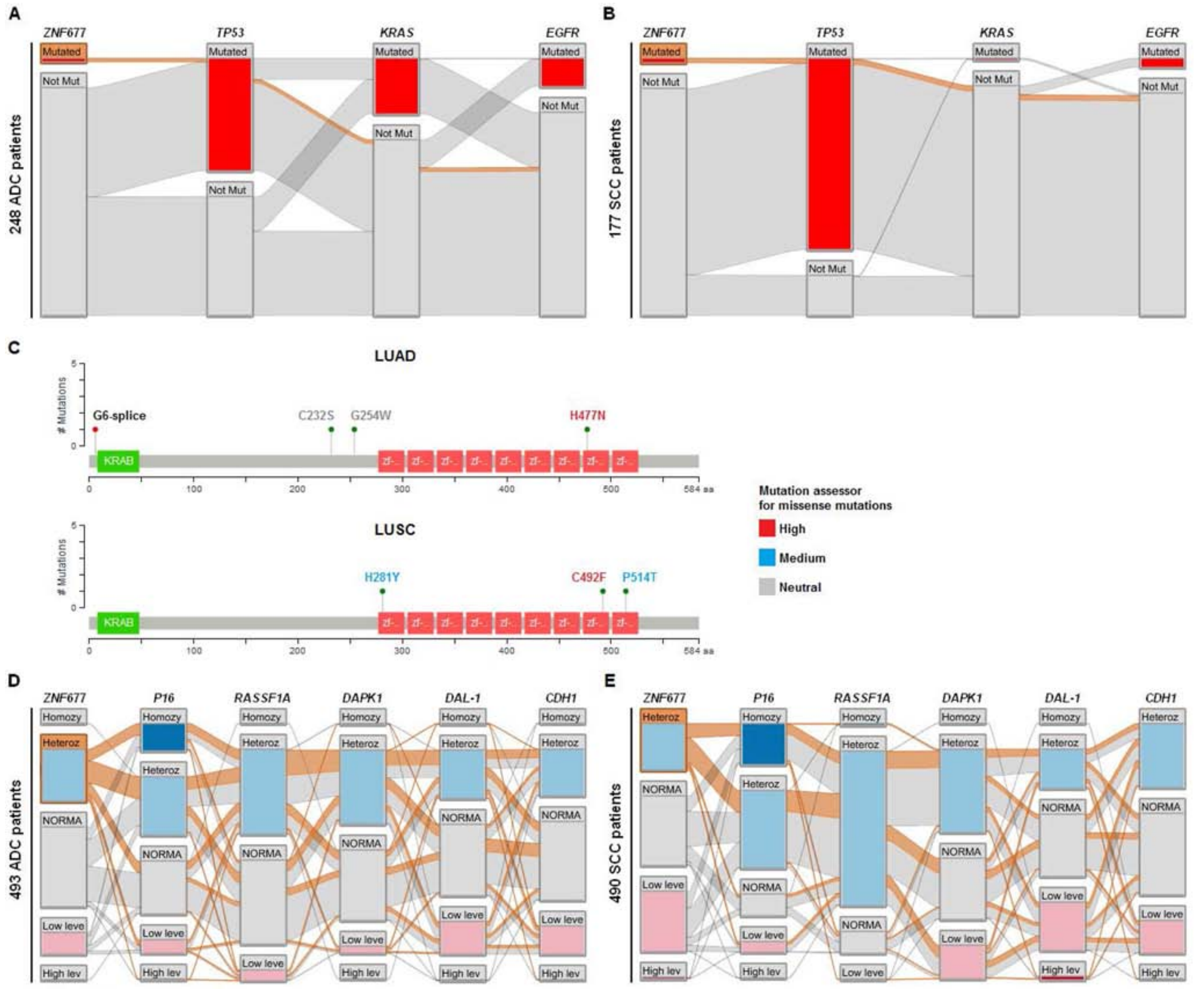

Supplementary Figure S3: A summary of *ZNF677* mutations (A-C) and *ZNF677* copy number changes (D-E) in NSCLC patients analysed using Caleydo software (version 15.01.2014) is shown. ADC, adenocarcinomas; SCC, squamous cell carcinomas.

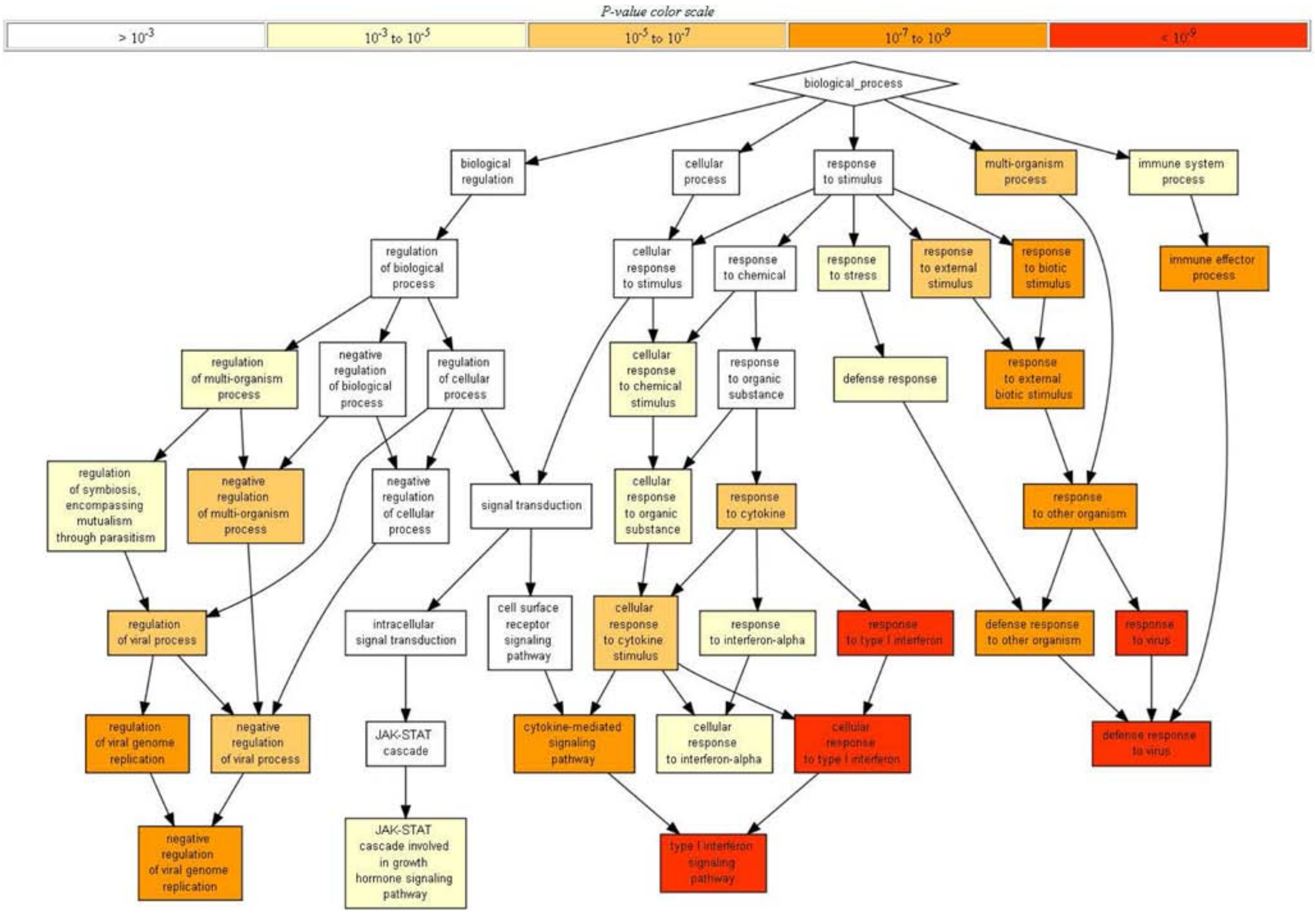

Supplementary Figure S4: Gene Ontology (GO) analysis regarding "biological process" of 34 ZNF677 regulated genes in NSCLC cells is shown. Statistically significant overrepresented GO categories are marked in colour (light yellow to red).

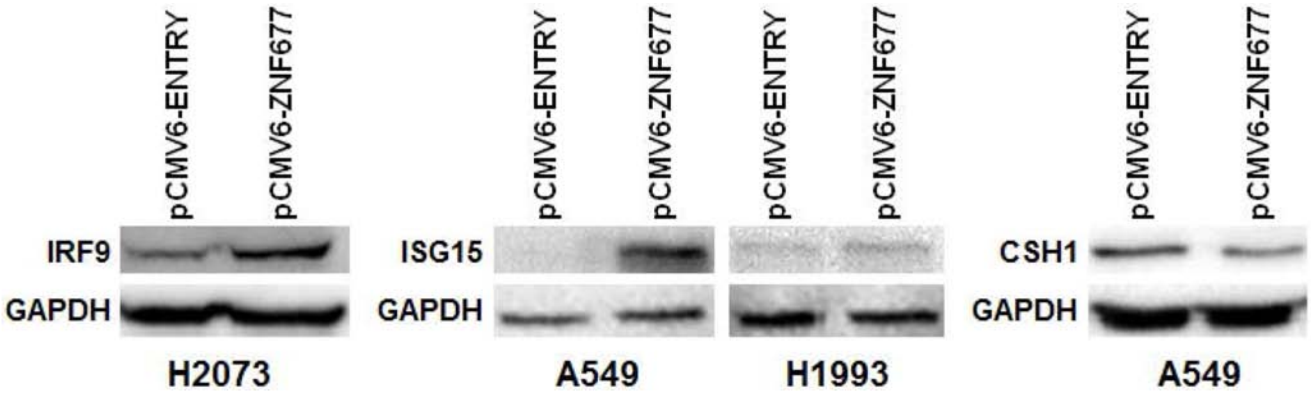

Supplementary Figure S5: Examples of Western blot analyses using lysates from pCMV6-ENTRY and pCMV6-ZNF677 transfected NSCLC cell lines and antibodies to IRF9, ISG15, CSH1 and GAPDH are shown.

Supplementary Table S1. Summary of ZNF677 regulated genes in the NSCLC cell lines A549, NCI-H1993 and NCI-H2073

A549 up	A549 down	NCI-H1993 up	NCI-H1993 down	NCI-H2073 up	NCI-H2073 down
AC015849.2	*AC093063.1*	*AF127577.11*	*AC017101.10*	*ALDH1A1*	*AC004258.1*
AC091304.2	*AKR1CL1*	*ALDH1A1*	*AC079354.5*	*AP001610.5*	*AC021066.1*
AC138749.1	*AL163636.6*	*AP001610.5*	*AC119673.1*	*C19orf33*	*AL928654.7*
AC139495.1	*ALDH1A3*	*APOL6*	*C3orf14*	*CD7*	*ARL14*
AC232323.1	*ANKRD1*	*C19orf66*	*CDKN3*	*CDKN1A*	*CRIP1*
ACVRL1	*APOH*	*C1orf170*	*CSH1*	*CMPK2*	*CSH1*
AD000671.1	*ARRDC4*	*CMPK2*	*CSH2*	*DDX58*	*CSH2*
AL078621.3	*ASNS*	*CXCL5*	*GH1*	*DTX3L*	*CTSD*
AL353997.5	*ATF3*	*DDX60*	*GH2*	*EIF2AK2*	*GH1*
AL356356.1	*BCYRN1*	*DTX3L*	*GPRIN3*	*GBP1*	*GH2*
APOBR	*BTG2*	*HERC6*	*GREB1L*	*HERC6*	*HBB*
BDKRB1	*C5*	*IFI44*	*GSTM1*	*HMOX1*	*HSPB1*
BX255923.3	*CD300LB*	*IFI44L*	*KRT3*	*IFI27*	*KISS1*
C15orf48	*CDH17*	*IFI6*	*LSM3*	*IFI44L*	*KRT6A*
C17orf96	*CLDN2*	*IFIH1*	*PFDN4*	*IFI6*	*KRT83*
C1QTNF1	*CNTN1*	*IFIT1*	*PHIP*	*IFIH1*	*KRT86*
C20orf141	*CSH1*	*IFIT2*	*PRR11*	*IFIT1*	*LYZ*
CCL17	*CSH2*	*IFIT3*	*RP11-532E4.2*	*IFIT1B*	*MT2A*
CCL3	*CYP1A1*	*IFIT5*	*RPS29*	*IFIT2*	*MUC4*
CCL3L1	*GEM*	*IRF9*	*RSL24D1*	*IFIT3*	*NDUFA11*
CCL3L3	*GH1*	*ISG15*	*SLC16A7*	*IFITM1*	*PTPRC*
CCL4	*GH2*	*KRT76*	*TAOK1*	*IL24*	*RP11-1143G9.4*
CCL4L1	*PCDH9*	*LAMP3*	*TNFSF15*	*IRF9*	*RP11-206L10.3*
CCL4L2	*PPM1D*	*MUC12*	*ZBED6*	*ISG15*	*RP11-34P13.12*
CCL5	*RGS4*	*MX1*	*ZDHHC20*	*KRT81*	*RPL28*
CD74	*RMND5A*	*OAS3*	*ZNF192*	*MMP13*	*RPS28*
CD82	*RNASE4*	*OASL*		*MX1*	*SLC9A3R2*
CHEK2	*RP11-326C3.10*	*PARP10*		*MX2*	
CHI3L2	*SLC38A1*	*PARP14*		*OAS1*	
CITED2	*STC2*	*PARP9*		*OAS2*	
CLDN4	*TM4SF20*	*RP11-468E2.4*		*OAS3*	
COL6A1	*TM4SF4*	*RP4-697K14.12*		*PARP12*	
CSAG2	*TNFRSF10D*	*RP4-697K14.7*		*PARP14*	
CSAG3	*ZNF77*	*RSAD2*		*PARP9*	
CSF2		*SAMD9L*		*PLSCR1*	
CSF3		*SP110*		*RN7SL1*	

(*Continued*)

A549 up	A549 down	NCI-H1993 up	NCI-H1993 down	NCI-H2073 up	NCI-H2073 down
CTD-2611K5.6		*TRANK1*		*RP11-259K21.3*	
CTSE		*TRIM21*		*RP11-336A10.2*	
CTSG		*UBE2L6*		*RP4-697K14.7*	
CX3CL1		*USP18*		*RPL36*	
CXCL10		*USP41*		*RSAD2*	
CXCL11		*ZNF321P*		*SAA1*	
DARC		*ZNF415*		*SAA2*	
DEFA1		*ZNF677*		*SAA2-SAA4*	
DEFA1B		*ZNF816*		*SAMD9*	
DEFA3				*SFN*	
DHX58				*SP110*	
DUOX2				*TDRD7*	
FAM131A				*TRANK1*	
FCGR3B				*UBA7*	
FZD4				*UBE2S*	
GABBR1				*XAF1*	
GALNT12				*ZNF321P*	
GBP5				*ZNF415*	
GOLGA6L1				*ZNF677*	
GOLGA6L6				*ZNF816*	
GPR84					
GREM1					
HBB					
HCAR2					
HCAR3					
HIP1R					
HRASLS2					
HSD11B1					
HSPG2					
HYDIN					
ICAM1					
IER5					
IFNB1					
IGFBP6					
IGFN1					
IL10RA					
IL28A					

(*Continued*)

A549 up	A549 down	NCI-H1993 up	NCI-H1993 down	NCI-H2073 up	NCI-H2073 down
IL28B					
IL29					
IL2RG					
IL6					
IL7R					
ISG15					
ISG20					
ITGA5					
KRT14					
KRT16					
KRT17					
KRT17P1					
LAMB3					
LAYN					
LRRC38					
LTF					
MARCKSL1					
MPO					
MSMB					
MUC12					
MUC16					
MUC2					
MUC4					
NAIF1					
NCF2					
NKX3-1					
NPTX1					
NUPR1					
OLR1					
PDC					
PDLIM7					
PDZK1IP1					
PIK3R5					
PLCG2					
PRG2					
PTAFR					

(*Continued*)

A549 up	A549 down	NCI-H1993 up	NCI-H1993 down	NCI-H2073 up	NCI-H2073 down
RAET1L					
RASSF5					
RCSD1					
REXO1L1					
RHCG					
RN7SL1					
RND1					
RP11-1286E23.12					
RP11-1286E23.14					
RP11-1286E23.15					
RP11-1286E23.16					
RP11-1286E23.17					
RP11-1286E23.18					
RP11-1286E23.5					
RP11-1286E23.6					
RP11-1286E23.7					
RP11-1286E23.8					
RP11-138B4.1					
RP11-143M1.3					
RP11-324E6.6					
RP11-356I2.2					
RP11-467N20.5					
RP11-519G16.5					
RP11-561O23.5					
RP11-58A12.2					
RP11-598F7.6					
RPPH1					
S100A8					
SAA2-SAA4					
SBSN					
SDC4					
SERPINA1					
SLC2A6					
SLC6A12					
STAT5A					
STK19					

(*Continued*)

A549 up	A549 down	NCI-H1993 up	NCI-H1993 down	NCI-H2073 up	NCI-H2073 down
TAC3					
TIMP1					
TMEM171					
TMEM173					
TMEM239					
TNF					
TNFAIP6					
TNFRSF8					
TNNT2					
TRAF1					
TRPA1					
USP17					
VANGL2					
ZBTB32					
ZC3H12A					
ZFP36					
ZFP36L2					
ZNF415					
ZNF677					

Supplementary Table S2. Summary of GO enrichment analyses

GO term	Description	RNA-seq		E-GEOD-18842		E-GEOD-19188	
		P-value	FDR	P-value	FDR	P-value	FDR
**Enriched in RNA-seq, E-GEOD-18842 and E-GEOD-19188**							
**GO:0051707**	Response to other organism	1.90E-09	3.43E-06	4.02E-05	3.05E-03	9.82E-11	9.87E-09
**GO:0043207**	Response to external biotic stimulus	2.77E-09	4.38E-06	7.56E-08	1.35E-05	1.67E-14	3.15E-12
**GO:0009607**	Response to biotic stimulus	4.23E-09	5.94E-06	3.95E-08	7.69E-06	4.91E-15	9.71E-13
**GO:0002252**	Immune effector process	1.44E-08	1.65E-05	4.08E-06	4.24E-04	3.47E-15	7.09E-13
**GO:0019221**	Cytokine-mediated signaling pathway	7.46E-08	7.26E-05	7.86E-05	5.53E-03	4.01E-10	3.53E-08
**GO:0071345**	Cellular response to cytokine stimulus	7.54E-07	6.36E-04	1.80E-06	2.02E-04	2.19E-12	2.80E-10
**GO:0034097**	Response to cytokine	3.20E-06	2.25E-03	9.26E-08	1.58E-05	9.26E-14	1.50E-11
**GO:0009605**	Response to external stimulus	6.19E-06	3.91E-03	2.78E-11	1.26E-08	5.09E-21	2.30E-18
**GO:0006952**	Defense response	1.57E-05	9.01E-03	1.04E-13	9.44E-11	1.27E-27	1.78E-24
**GO:0002376**	Immune system process	5.00E-05	2.75E-02	3.51E-21	1.48E-17	6.00E-43	2.53E-39
**GO:0006950**	Response to stress	1.52E-04	7.37E-02	3.12E-06	3.29E-04	6.77E-16	1.43E-13
**GO:0070887**	Cellular response to chemical stimulus	1.68E-04	7.87E-02	2.25E-08	4.60E-06	1.46E-26	1.32E-23
**GO:0071310**	Cellular response to organic substance	2.02E-04	9.12E-02	1.41E-05	1.27E-03	8.72E-23	5.02E-20
**Enriched in RNA-seq and E-GEOD-19188**							
**GO:0034340**	Response to type I interferon	1.69E-13	7.11E-10			9.33E-08	5.52E-06
**GO:0060337**	Type I interferon signaling pathway	1.48E-13	9.38E-10			6.18E-08	3.84E-06
**GO:0071357**	Cellular response to type I interferon	1.48E-13	1.88E-09			6.18E-08	3.86E-06
**GO:0051607**	Defense response to virus	1.42E-12	4.48E-09			1.55E-04	4.48E-03
**GO:0009615**	Response to virus	5.26E-12	1.33E-08			5.64E-08	3.59E-06
**GO:0098542**	Defense response to other organism	1.56E-09	3.30E-06			2.02E-07	1.11E-05
**GO:0051704**	Multi-organism process	2.84E-06	2.11E-03			4.08E-05	1.43E-03

Primary tumor samples from datasets E-GEOD-18842 and E-GEOD-19188 were grouped in "*ZNF677* low expressing" and in "*ZNF677* high expressing" tumors using the 25th quartile of *ZNF677* expression as cut-off level. Differentially expressed genes, identified by SAM analysis, were applied to GO enrichment analyses.
